# Maternal and Perinatal Outcomes in Pregnancies Complicated by Predominantly Benign Ovarian Lesions: A Retrospective Case-Control Study of 305 Pregnancies

**DOI:** 10.3390/cancers18213463

**Published:** 2026-10-27

**Authors:** Aneta Zamojska, Monika Kopeć, Jan Kotarski, Katarzyna Dos Santos Szewczyk, Marek Gogacz

**Affiliations:** 1 Department of Obstetrics and Perinatology, Hospital “Inflandzka”, 00-189 Warsaw, Poland; 2 Department of Obstetrics, Gynecology and Pregnancy Pathology, Independent Public Hospital in Łuków, 21-400 Łuków, Poland; 3 Independent Laboratory of Cancer Diagnostics and Immunology, Department of Oncological Gynaecology and Gynaecology, Faculty of Medicine, Medical University of Lublin, Chodźki 1, 20-093 Lublin, Poland; 4 Department of Pharmaceutical Botany, Medical University of Lublin, Chodźki 1, 20-093 Lublin, Poland; k.szewczyk@umlub.pl; 5 Chair and Department of Gynecology, Medical University of Lublin, 20-059 Lublin, Poland; marek.gogacz@umlub.edu.pl

**Keywords:** lesion, benign ovarian neoplasm, adnexal mass, pregnancy, perinatal outcomes, caesarean section, atrial septal defect, case–control study

## Abstract

Background: Ovarian lesions during pregnancy pose diagnostic and therapeutic challenges for clinicians. This study aimed to describe the characteristics of ovarian lesions diagnosed during pregnancy and compare maternal and perinatal outcomes with those of pregnancies without ovarian pathology. Methods: A retrospective case–control study was conducted on 305 pregnant women hospitalized at the Department of Obstetrics and Perinatology in Lublin (2011–2020). The study group comprised 105 women with benign ovarian neoplasms (ICD-10 D27; *n* = 102) or ovarian cancer (C56; *n* = 3). The reference group comprised 200 women without ovarian pathology, selected by simple random sampling from hospital records. The main outcomes were mode of delivery, duration of pregnancy, birth weight, and APGAR scores. Comorbidities, neonatal diagnoses, and correlations were analyzed as exploratory outcomes using the false discovery rate (FDR) correction. Results: Maternal age did not differ between the groups (*p* = 0.242), whereas BMI was lower in the study group (26.4 vs. 28.2 kg/m^2^; *p* = 0.001), but not among women who delivered in the department (*p* = 0.777). Caesarean delivery was recorded in 57/58 women who delivered in the department (98.3%) vs. 97/200 (48.5%) in the reference group (*p* < 0.001). In the worst-case sensitivity analysis (*n* = 99), the proportion was 57.6% (*p* = 0.143). The gestational age at delivery was slightly lower in the study group than in the reference group (38.2 vs. 38.8 weeks; *p* = 0.009). Overall, 42% of the lesions were first detected during the caesarean section. Birth weight (*p* = 0.696) and APGAR scores (*p* = 0.418) did not differ. Among newborns with available records (48 vs. 198), atrial septal defects were more frequent in the study group (22.9% vs. 6.1%; RR 3.78, 95% CI 1.78–8.04; FDR-adjusted *p* = 0.022). Lesion size was weakly correlated with the number of maternal comorbidities (rho = 0.247; *p* = 0.016). Conclusions: In this cohort of predominantly benign ovarian lesions, birth weights and APGAR scores were comparable to those of the reference group. The high caesarean rate largely reflects intraoperative lesion detection and incomplete data rather than the effect of the lesion itself, and the neonatal findings are hypothesis-generating.

## Simple Summary

Ovarian lesions diagnosed during pregnancy are a clinical concern, partly because of the global trend of women delaying childbearing, which increases the likelihood of encountering benign and, less often, malignant ovarian lesions alongside pregnancy. We conducted a retrospective case–control study of 305 pregnancies, comparing 105 women with an ovarian lesion (benign ovarian neoplasm or ovarian cancer; 97.1% benign) with 200 randomly sampled pregnant women without ovarian pathology to describe maternal and perinatal outcomes. Birth weight and APGAR scores did not differ between the groups. Caesarean delivery was more frequent among women with ovarian lesions, but this finding was strongly influenced by the high proportion of lesions detected incidentally at caesarean section and by missing delivery data. A higher frequency of neonatal atrial septal defects was observed in the subset with available neonatal records. These exploratory findings may help clinicians counsel pregnant women with ovarian lesions and should be confirmed in prospective studies.

## 1. Introduction

The impact of pathological ovarian lesions on pregnancy and perinatal outcomes remains a broad and relevant topic. Changing dietary habits, levels of physical activity, stress, low public awareness of cancer, geographical latitude, and the surrounding environment entail dramatic changes in the choice of an appropriate diagnostic and therapeutic pathway by a mother-to-be diagnosed with an ovarian lesion, who already faces many dilemmas relating to the life and wellbeing of her child [1,2,3]. The medical team involved in the care of a pregnant woman with an ovarian lesion faces a challenge in terms of adequate diagnosis and appropriate therapy, and dilemmas in assessing the advantages and disadvantages of medical management, access to modern diagnostic methods, and the experience available, so that the measures taken are the best possible for the present moment. Cancer within the ovary in pregnant women presents not only an ethical dilemma but also a clinical one. Pregnancy-associated malignancy is defined as cancer diagnosed during pregnancy or within 12 months after delivery or miscarriage [4,5].

**Figure 1:**
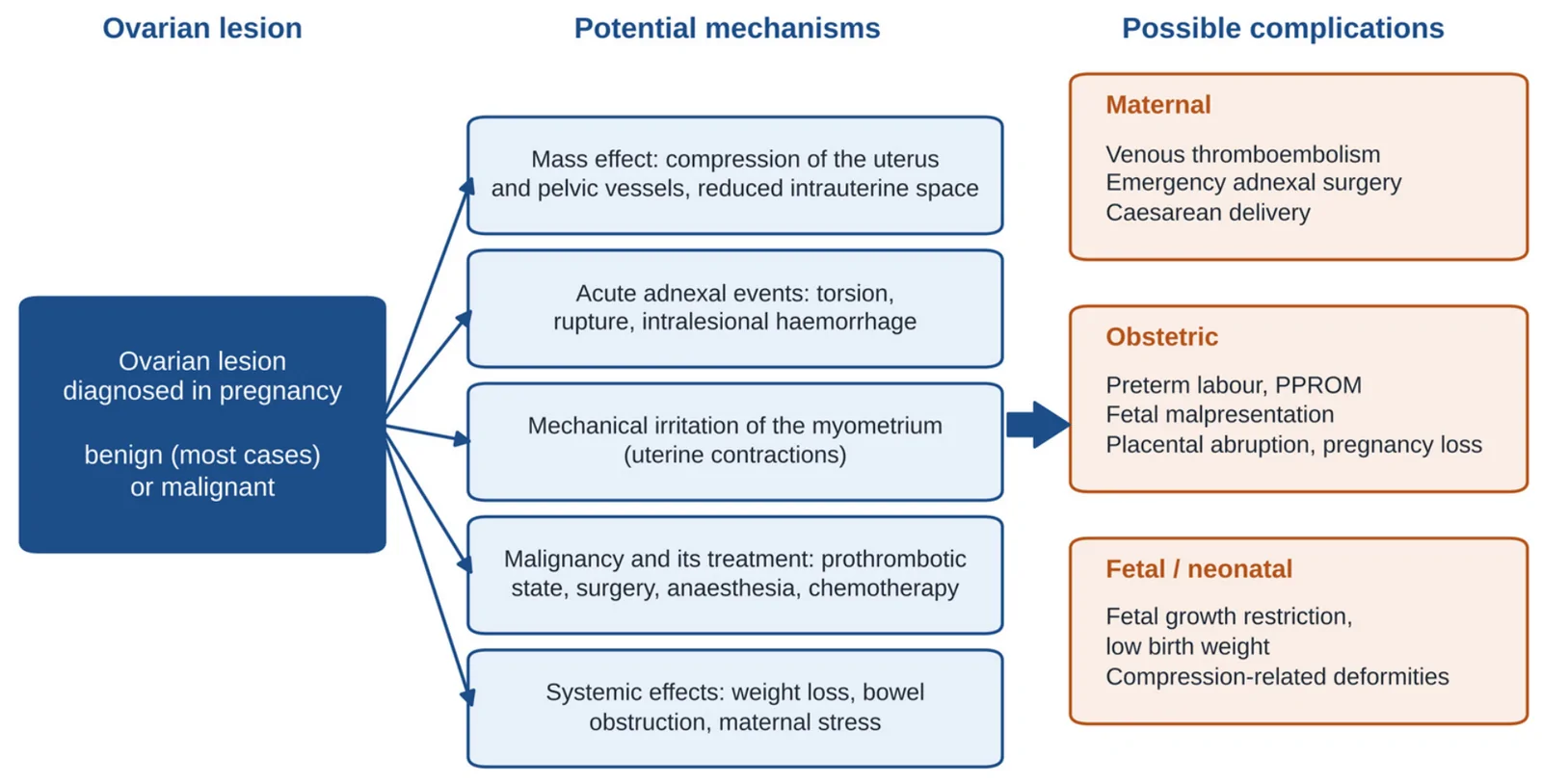
Schematic overview of the potential mechanisms through which an ovarian lesion may affect the course of pregnancy and the maternal, obstetric, and fetal/neonatal complications described in the literature [6,8,9,10,11].

The impact of an ovarian lesion on pregnancy development is not only due to the presence of the lesion itself but is also indirectly linked to the treatment strategy adopted, the anesthetic used for the operation, or the permanent stress experienced by the mother [6]. According to a study by Singh et al. (2025), among numerous malignancies, the reported prevalence of ovarian tumors in pregnancy varies widely across the literature, historically cited as 0.15–5.7% [7]. However, modern prospective ultrasound-based cohorts indicate a considerably lower incidence of approximately 1–2% of pregnancies, of which only about 1–6% of detected masses prove malignant [1,3]. Patients who have lost weight and appetite during the course of cancer are at greater risk of not having an adequate caloric supply for the developing fetus, which may result in a low birth weight baby. The presence of an ovarian lesion of significant size can result in fetal malposition and the completion of pregnancy by surgical delivery [8]. Pregnancy increases the risk of thromboembolism through changes in the coagulation system, such as a decrease in protein S, an increase in fibrinolysis inhibitor activity, a decrease in factor XI and factor XIII, and an increase in fibrinogen [9]. The presence of ovarian malignancy and the chosen treatment also has a strong prothrombotic effect. An increase in the stage of any cancer increases the risk of thromboembolism and, consequently, pulmonary embolism. Ovarian lesions of significant size can cause disruption of anatomical relationships and limit the environment for the developing fetus. Reduction in this space by pressure of the ovarian lesion on the uterus from outside may increase the likelihood of deformities in the child [10]. By exerting pressure on the uterine muscle, large ovarian lesions can also stimulate the uterus to contract and initiate preterm labor [11]. In addition, a lesion increasing in size may press on the internal iliac arteries, which supply the uterine muscle with blood through the uterine artery, and impair the blood supply to the uterus, thus adversely altering the environment in which the fetus develops. In their study, Gholson et al. (2014) reported that infants of mothers with large adnexal masses during pregnancy had a lower birth weight than those of mothers without such masses; among 13,677 live-born babies, the mean birth weight was 2944 g in the study group and 3169 g in the control group [11,12]. Ovarian tumors, especially in advanced stages, by exerting pressure on the rectum and infiltrating the wall of the intestinal loops, can cause intestinal obstruction in the mother, thus leading to water and electrolyte disturbances in the fetus. Pregnancy itself does not adversely affect the course of ovarian cancer. The literature emphasizes that a multidisciplinary team of specialists represented by an obstetrician, gynecologist, oncologist, neonatologist, psychiatrist, oncology psychologist, and pathologist is essential for the care of a pregnant woman diagnosed with an ovarian malignant tumor. Fetal well-being in this group of pregnant women should be frequently and carefully monitored to ensure optimal time and conditions for the birth of the baby [13,14,15]. The potential mechanisms and complications are summarized schematically in Figure 1.

Despite the growing body of literature on ovarian tumors during pregnancy, most published series are relatively small, originate from tertiary oncology referral centers, and focus predominantly on malignant lesions, which account for only a small proportion of cases encountered in routine obstetric practice. There is still a relative paucity of studies conducted in general obstetric settings that directly compare the complete clinical course, including diagnosis, delivery outcomes, and neonatal well-being, of women with predominantly benign ovarian lesions with that of a contemporaneous reference population of pregnant women without ovarian pathology managed at the same institution.

The present retrospective case–control study was designed to address this gap by analyzing 305 pregnancies managed over a 10-year period at a single Polish obstetric referral center, including 105 pregnancies complicated by an ovarian lesion and 200 reference pregnancies. This study aimed to describe maternal comorbidities, mode of delivery, and neonatal outcomes in this population and to generate hypotheses for future prospective studies on risk stratification and counselling of pregnant women with ovarian lesions, particularly in the context of the increasing age at first pregnancy.

## 2. Materials and Methods

This was a retrospective, single-center case–control study based on archival medical records. It is reported with reference to the STROBE (Strengthening the Reporting of Observational Studies in Epidemiology) statement for case–control studies.

### 2.1. Study Design, Setting, and Participants

**Figure 2:**
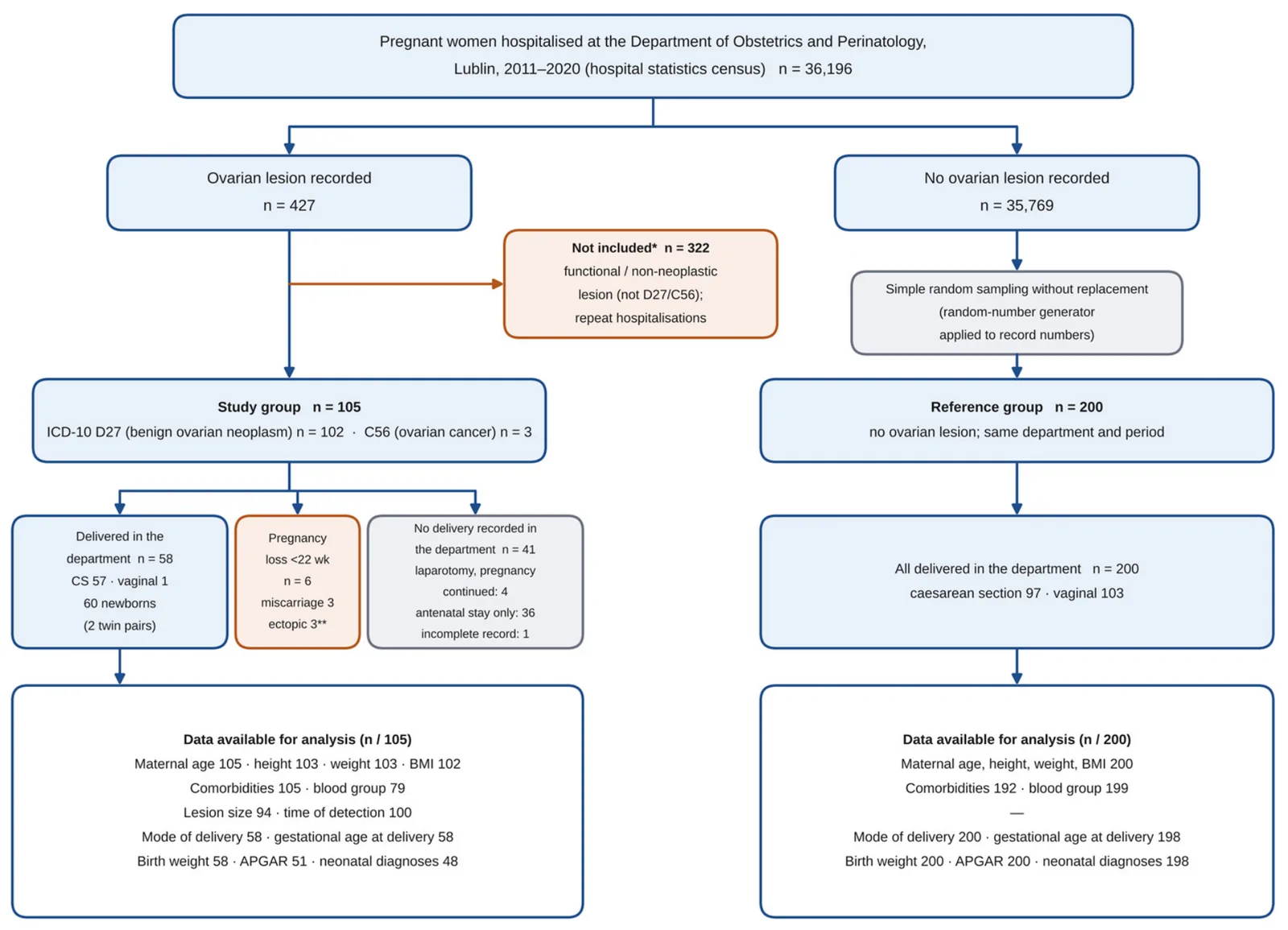
Flow diagram of the selection of the study and reference groups, pregnancy outcomes in the study group, and the number of women with available data for each variable (for birth weight and APGAR score in the study group, the number of newborns). * Lesions coded as functional or other non-neoplastic adnexal lesions (not ICD-10 D27/C56) and repeat hospitalizations of women already included (one index hospitalization per woman). ** Tubal ectopic pregnancies treated surgically. CS, caesarean section.

This study included 305 pregnant women hospitalized at the Department of Obstetrics and Perinatology in Lublin between 2011 and 2020. The sampling frame was the census of hospitalizations maintained by the hospital statistics department, which comprised 36,196 pregnant women in this period; an ovarian lesion was recorded in 427 of them (Figure 2). The study group comprised 105 pregnant women with a diagnosis of benign neoplasm of the ovary (ICD-10 D27) or malignant neoplasm of the ovary (ICD-10 C56), regardless of age. The diagnosis could be the main or a coexisting diagnosis and could be established before, during, or at the end of pregnancy, including intraoperatively at caesarean section. The remaining 322 women with a recorded ovarian lesion were not included because the lesion was coded as a functional or other non-neoplastic adnexal lesion (e.g., ICD-10 N83) and thus did not meet the D27/C56 criterion, or because the record was a repeat hospitalization of a woman already included (Figure 2). All eligible D27/C56 cases identified in the census were included. Each woman was included only once (one index hospitalization). The reference group consisted of 200 pregnant women without any recorded ovarian lesion who were hospitalized in the same department during the same period. Reference women were selected by simple random sampling without replacement: record numbers were drawn with a random-number generator from the list of eligible hospitalizations, and a woman once drawn could not be drawn again. No matching was performed. Approximately two reference women were sampled per case to increase the precision of between-group comparisons. No formal a priori sample size calculation was performed: the size of the study group was determined by the number of eligible cases identified in the census, and the statistical software was used only to draw the random sample of reference women. With 105 cases and 200 reference women, the study had 80% power (two-sided α = 0.05) to detect a standardized mean difference of 0.34 between the groups. No randomization in the sense of allocation to an intervention took place: group membership was determined by the presence or absence of an ovarian lesion.

Terminology. Throughout the manuscript, “ovarian lesion” is used as an umbrella term for all lesions fulfilling the inclusion criteria (ICD-10 D27 or C56), that is, benign ovarian neoplasms and ovarian cancers, irrespective of their cystic, solid, or mixed morphology. “Lesion size” refers to the largest diameter of the lesion in centimeters, as measured on ultrasound. The term “ovarian cyst” is used only when citing literature on cystic lesions. On histopathological examination, 102 lesions (97.1%) were benign, and 3 (2.9%) were malignant. The benign lesions comprised 89 simple cysts, 8 mature cystic teratomas, and 5 endometriotic cysts, and the malignant lesions comprised 2 mucinous adenocarcinomas and 1 serous carcinoma; 3 lesions were solid (see Section 3).

### 2.2. Variables and Outcome Definitions

The following variables were extracted from the medical records: maternal age, height, body weight, body mass index (BMI; categorized as <25.0, 25.0–29.9, and ≥30.0 kg/m^2^), blood group, comorbidities, number of pregnancies, duration of pregnancy, mode of delivery (vaginal or caesarean), postpartum hemoglobin concentration (postpartum anemia defined as hemoglobin ≤ 11 g/dL), pregnancy loss, and the size and time of detection of the ovarian lesion. The time of detection was classified as the first (≤14 weeks), second (15–27 weeks), or third trimester (≥28 weeks), or as detected intraoperatively during caesarean section. Neonatal outcomes comprised birth weight, the APGAR score at 1 min, categorized as poor (0–3), moderate (4–7), or good (8–10), and neonatal diagnoses coded according to ICD-10 in the neonatal records; a newborn could have more than one diagnosis. Fetal echocardiography was performed by a certified fetal cardiology specialist at 22–24 weeks of gestation according to the recommendations of the Polish Society of Gynecologists and Obstetricians. Atrial septal defect was diagnosed on postnatal echocardiography performed in the neonatology department of the University Hospital, which distinguished secundum atrial septal defect from patent foramen ovale. Preterm birth was defined as delivery before 37 + 0 weeks of gestation, very preterm birth as delivery before 32 + 0 weeks, and extremely preterm birth as delivery before 28 + 0 weeks; pregnancy loss before 22 + 0 weeks was recorded separately. Duration of pregnancy was defined as gestational age at delivery in completed weeks and was analyzed for women who delivered in the department.

Mode of delivery, duration of pregnancy (including preterm birth), birth weight, and APGAR score were designated as the main outcomes. Maternal comorbidities, neonatal diagnoses, blood group distribution, and correlations between lesion characteristics and clinical parameters were regarded as secondary exploratory analyses. The following relationships were explored in the study group:

- Lesion size and maternal age;
- Maternal age and gestational age at the time of delivery;
- Gestational age at delivery and newborn’s APGAR score;
- Lesion size and APGAR score;
- Lesion size and number of maternal comorbidities;
- Number of pregnancies and postpartum anemia;
- Lesion size and postpartum anemia;
- Lesion size and time of detection (trimester or intraoperative);
- Lesion size and number of pregnancies.

Data were extracted from the medical records stored in the Central Hospital Archive based on the ICD-10 codes assigned to each hospitalization. Histopathological examination was performed on tissue obtained surgically, in most cases, at the time of caesarean section.

### 2.3. Missing Data

All analyses were performed on women with available data for the respective variables (complete-case analysis), and the number of women with available data is reported for every variable (Figure 2 and Section 3). Missing data were due to incomplete archival records. Of the 105 women in the study group, 58 delivered in the department (57 by caesarean section and 1 vaginally; 60 newborns, including two twin pairs). No delivery was recorded in the department for the remaining 47 women: 6 had a pregnancy loss before 22 weeks (three miscarriages and three tubal ectopic pregnancies treated surgically), 4 underwent laparotomy for an adnexal mass during pregnancy and continued the pregnancy, 36 were hospitalized only antenatally (mostly in the first or second trimester) and delivered elsewhere or their delivery records were not available, and in 1 case the documentation was incomplete. Neonatal outcomes were therefore available only for newborns delivered in the department, and neonatal diagnoses were recorded for 48 of the 58 women who delivered in the department. Because missingness was concentrated in the study group and is unlikely to be completely random, the robustness of the main finding on the mode of delivery was assessed in a sensitivity analysis, assuming that all women without a recorded delivery, excluding those with a pregnancy loss before 22 weeks, delivered either vaginally (worst-case scenario) or by caesarean section (best-case scenario). No imputation was performed for missing data.

### 2.4. Statistical Analysis

Statistical analysis was performed using IBM SPSS Statistics (version 25; IBM Corp., Armonk, NY, USA). Continuous variables are presented as mean and standard deviation (SD), median, quartiles (Q1–Q3), and range, and categorical variables as number and percentage of women with available data. Means were compared using Student’s *t*-test for independent samples or, when variances were unequal, Welch’s *t*-test. Categorical variables were compared using the chi-square (χ^2^) test or, when more than 20% of expected cell counts were below 5, Fisher’s exact test (Fisher–Freeman–Halton test for tables larger than 2 × 2, with Monte Carlo estimation based on 100,000 samples where needed). Relationships between categorical variables are described as associations. For binary outcomes, relative risks (RR) with 95% confidence intervals (CI) were calculated (with Haldane correction for zero cells), and for continuous outcomes, mean differences with 95% CIs. Correlations between continuous or ordinal variables were assessed using Spearman’s rank correlation coefficient (rho). Comorbidities were analyzed per patient: for each diagnostic category, the number of women with at least one diagnosis in that category was compared between the groups. Because of the large number of secondary comparisons, *p*-values for comorbidity categories and neonatal diagnoses were adjusted using the Benjamini–Hochberg false discovery rate (FDR) procedure, and adjusted values (q) are reported; all secondary analyses are considered exploratory. Multivariable regression was considered but was not performed for the mode of delivery, because only one vaginal delivery occurred among women with available data in the study group (quasi-complete separation), nor for neonatal diagnoses, because the small number of events (e.g., 11 cases of atrial septal defect in the study group) did not allow stable adjustment for more than one covariate. Because of its skewed distribution, gestational age at delivery was additionally compared using the Mann–Whitney U test. No formal a priori sample size calculation was performed (Section 2.1). A two-sided *p* < 0.05 was considered statistically significant.

## 3. Results

The study included 305 pregnant women: 105 (34.4%) in the study group and 200 (65.6%) in the reference group. The selection process and number of women with available data for each variable are shown in Figure 2.

The baseline maternal characteristics are summarized in Table 1. The mean age was 31.0 ± 5.1 years in the study group and 30.3 ± 5.0 years in the reference group (overall 30.5 years; range 18–45 years), and the difference was not statistically significant (t = −1.172, df = 303; *p* = 0.242). Mean height did not differ between the groups (166.8 vs. 166.3 cm; *p* = 0.457). Mean body weight was lower in the study group (73.1 vs. 77.9 kg; mean difference −4.8 kg, 95% CI −7.9 to −1.7; t = 3.098, df = 301; *p* = 0.002), as was mean BMI (26.4 vs. 28.2 kg/m^2^; mean difference −1.8 kg/m^2^, 95% CI −2.9 to −0.7; t = 3.268, df = 300; *p* = 0.001); the overall mean BMI was 27.6 kg/m^2^ (range 18.3–50.2 kg/m^2^). Because body weight in women without a recorded delivery was measured at an antenatal, mostly early-pregnancy hospitalization, the comparison was repeated for women who delivered in the department; in this subgroup, BMI did not differ from that in the reference group (28.3 ± 5.0 vs. 28.2 ± 4.1 kg/m^2^; *n* = 57/200; *p* = 0.777). The distribution of BMI categories differed between the groups (χ^2^ = 13.379, df = 2; *p* = 0.001), with a higher proportion of women with BMI < 25.0 kg/m^2^ in the study group (43.1% vs. 23.0%). The number of pregnancies (χ^2^ = 1.723, df = 3; *p* = 0.632) and the ABO and Rh blood group distributions did not differ between the groups; ARh+ and 0Rh+ were the most common blood groups in both groups (Table S1).

Maternal comorbidities analyzed per patient are shown in Table 2. At least one comorbidity was recorded in all women in the study group and in 136 of 192 women (70.8%) in the reference group with available data. After FDR correction, genitourinary diseases (100.0% vs. 55.2%; q < 0.001), endocrine diseases (15.2% vs. 0.5%; q < 0.001), and metabolic diseases (6.7% vs. 0.0%; q = 0.002) were more frequent in the study group, whereas the differences in cardiovascular diseases (18.1% vs. 10.4%; q = 0.097), gastrointestinal diseases (6.7% vs. 1.6%; q = 0.074), immune diseases (4.8% vs. 13.0%; q = 0.063), and a history of caesarean section (16.2% vs. 8.3%; q = 0.081) were not statistically significant. Because the ovarian lesion itself may have been coded within the genitourinary chapter, the difference in genitourinary diseases should be interpreted with caution. A woman with several diagnoses within the same category was counted once. Therefore, the number of category assignments in the study group (178) is lower than the number of individual diagnoses recorded in the medical records.

The lesion characteristics are presented in Table 3. The lesion size was available for 94 women; the mean size was 4.4 ± 3.4 cm (median 3.5 cm; Q1–Q3 2.0–6.0 cm; range 0.5–20.0 cm). Lesions ≤ 2.5 cm, >2.5–5.0 cm, and >5.0 cm accounted for 31.9%, 34.0%, and 34.0% of cases, respectively. Of the 100 lesions with a known time of detection, 41 (41.0%) were detected in the first trimester, 13 (13.0%) in the second trimester, 4 (4.0%) in the third trimester, and 42 (42.0%) intraoperatively during caesarean section. On histopathological examination, 102 lesions (97.1%) were benign and 3 (2.9%; 95% CI 1.0–8.1%) were malignant; the three women with ovarian cancer delivered at 27, 35, and 36 weeks of gestation. Because of the small number of malignant cases, no separate statistical analysis was performed for this subgroup.

**Figure 3:**
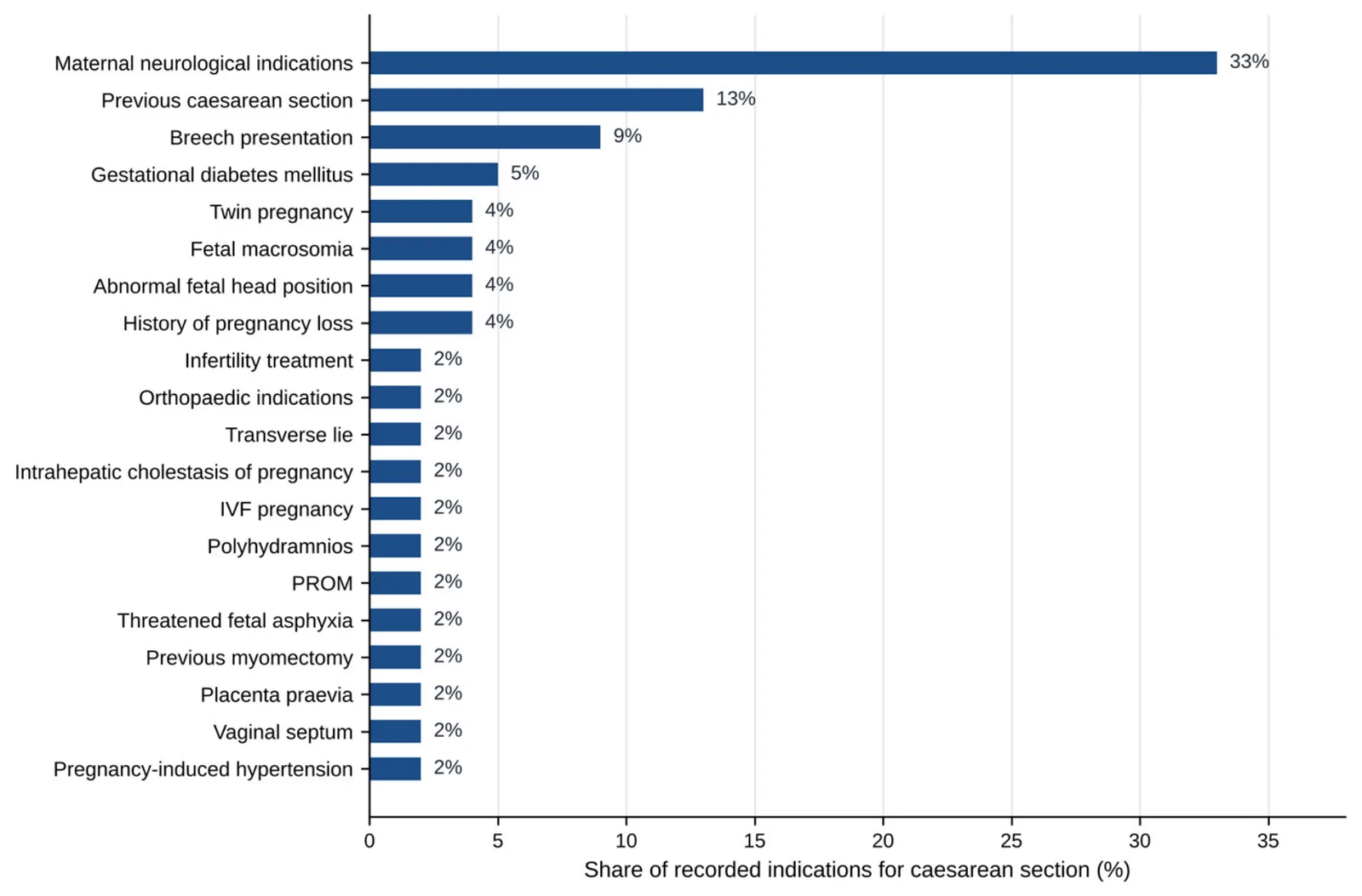
Recorded indications for caesarean section in the study group, expressed as a percentage of all recorded indications.

The pregnancy and delivery outcomes are presented in Table 4. A delivery in the department was recorded for 58 of 105 women (55.2%) in the study group and for all 200 women in the reference group (reasons for missing delivery data are given in Section 2.3 and Figure 2); eight women in the study group who underwent non-obstetric surgery during pregnancy (laparotomy for an adnexal mass or surgery for an ectopic pregnancy) were not counted as caesarean deliveries. Among the women who delivered, 57 of 58 women (98.3%; 95% CI 90.9–99.7%) in the study group and 97 of 200 women (48.5%) in the reference group underwent caesarean sections (Fisher’s exact test *p* < 0.001; RR 2.03, 95% CI 1.75–2.35). Importantly, 42 of the 100 lesions with a known time of detection were first identified intraoperatively during caesarean section; these women could, by definition, enter the study group only after a caesarean delivery, which inflates the caesarean rate in the study group. In the worst-case sensitivity analysis, in which all 41 women with an ongoing pregnancy but no recorded delivery were assumed to have delivered vaginally (*n* = 99; six pregnancy losses excluded), the caesarean rate in the study group was 57.6% (57/99; RR 1.19, 95% CI 0.95–1.48; *p* = 0.143). In the best-case scenario, it was 99.0% (98/99). When the analysis was restricted to women who delivered in the department and whose lesion had been detected antenatally, 15 of 16 women (93.8%; RR 1.93, 95% CI 1.60–2.34; *p* < 0.001) underwent caesarean sections. The recorded indications for caesarean section in the study group are shown in Figure 3; the most frequent were maternal neurological indications (33% of recorded indications), previous caesarean section (13%), and breech presentation (9%), whereas threatened fetal asphyxia accounted for 2% of indications.

Gestational age at delivery was available for 58 women who delivered in the department and for 198 women in the reference group. The mean gestational age at delivery was 38.2 ± 1.5 weeks in the study group and 38.8 ± 1.6 weeks in the reference group (mean difference −0.6 weeks, 95% CI −1.0 to −0.2; Welch’s t = 2.665, df = 99.8; *p* = 0.009; Mann–Whitney U test *p* < 0.001). Preterm birth (<37 + 0 weeks) occurred in 6 of 58 women (10.3%) in the study group and in 8 of 198 women (4.0%) in the reference group (RR 2.56, 95% CI 0.93–7.08; *p* = 0.094). Very preterm birth (<32 + 0 weeks) occurred in 0 and 2 women and extremely preterm birth (<28 + 0 weeks) in 0 and 1 woman, respectively. Pregnancy loss before 22 weeks occurred in 6 of the 105 women (5.7%) in the study group (three miscarriages and three tubal ectopic pregnancies). Postpartum anemia (hemoglobin ≤ 11 g/dL) was recorded in 43 of 94 women (45.7%) in the study group with available data.

Neonatal outcomes are presented in Table 5. Birth weight data were available for 58 newborns (from 56 deliveries, including two twin pairs) in the study group and 200 in the reference group; the mean birth weight was 3360 ± 530 g and 3387 ± 445 g, respectively (mean difference −27 g, 95% CI −164 to 109 g; t = −0.392, df = 256; *p* = 0.696), and the overall mean was 3381 g (range 1220–4500 g). APGAR scores were available for 51 and 200 newborns, respectively; 96.1% and 98.0% of newborns were in good condition (8–10 points), and the distribution of APGAR categories did not differ between the groups (Fisher–Freeman–Halton test *p* = 0.418). The distribution of individual APGAR scores is given in Table S2.

Neonatal diagnoses were available for 48 of the 58 women who delivered in the department (45.7% of the study group) and for 198 of the 200 newborns (99.0%) in the reference group. Among newborns with available records, atrial septal defect was recorded more frequently in the study group (11/48, 22.9% vs. 12/198, 6.1%; RR 3.78, 95% CI 1.78–8.04; *p* = 0.001; q = 0.022). Respiratory failure of the newborn (10.4% vs. 2.0%; RR 5.16, 95% CI 1.44–18.48; *p* = 0.016) and the diagnosis of ‘infant of a mother with gestational diabetes’ (8.3% vs. 0.5%; *p* = 0.006) were also more frequent in the study group, but these differences did not remain significant after FDR correction (q = 0.100 and q = 0.053, respectively). Unspecified neonatal jaundice occurred at a similar frequency in both groups (18.8% vs. 17.2%; *p* = 0.833). A complete list of neonatal diagnoses is provided in Table S3. Because neonatal records were missing for more than half of the study group, these comparisons were exploratory.

### Correlation and Association Analyses in the Study Group

**Figure 4:**
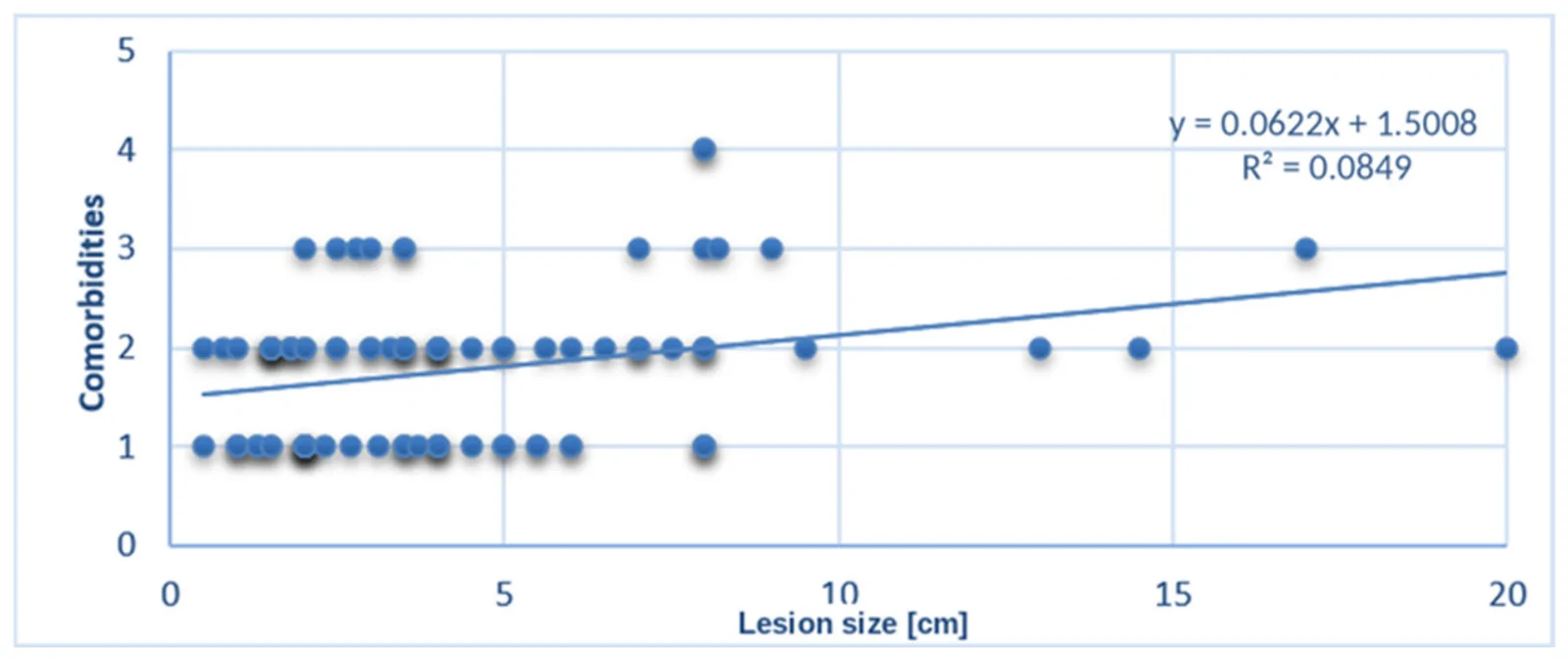
Lesion size and number of maternal comorbidities in the study group (Spearman’s rho = 0.247; *n* = 94; *p* = 0.016). The line indicates a linear trend.

**Figure 5:**
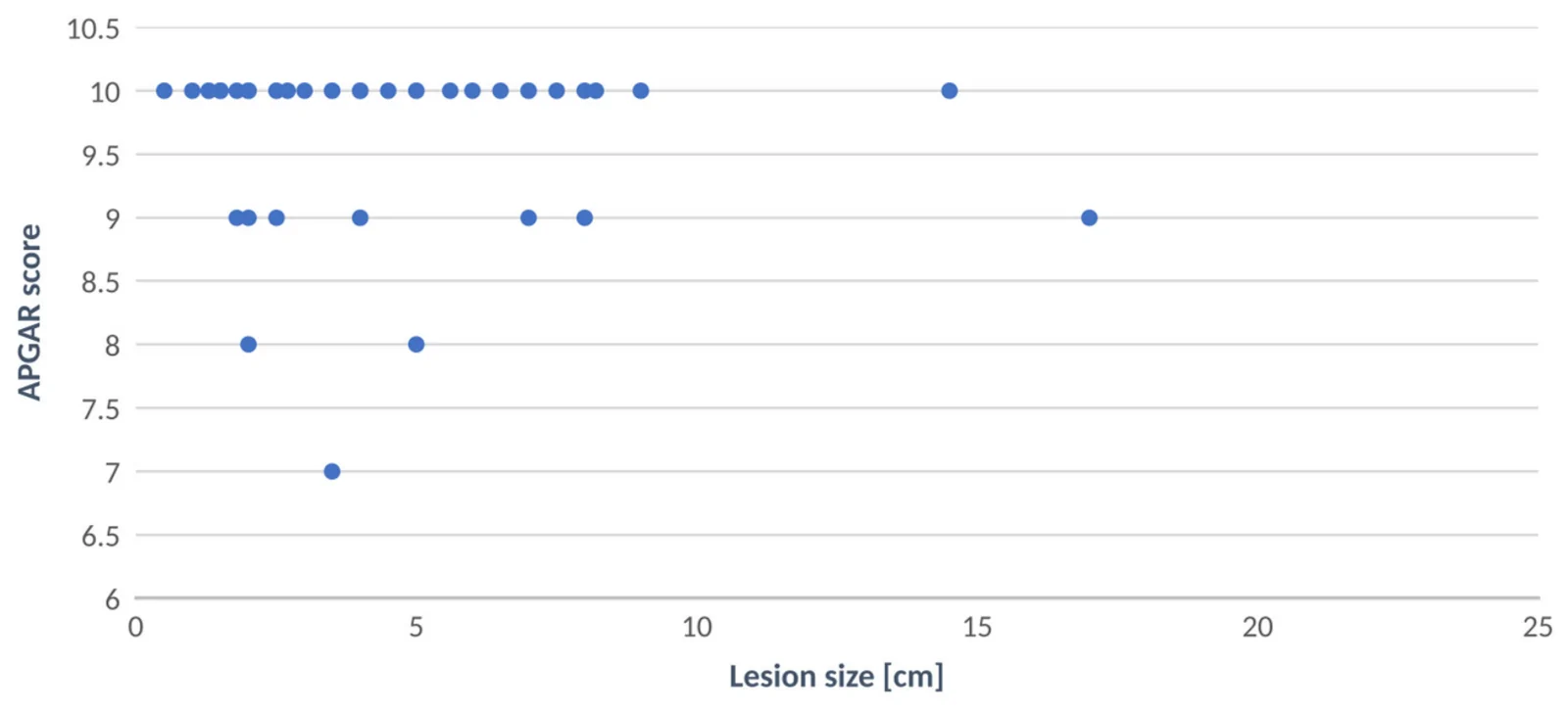
Lesion size and APGAR score in the study group (rho = −0.147; *n* = 50; *p* = 0.307).

**Figure 6:**
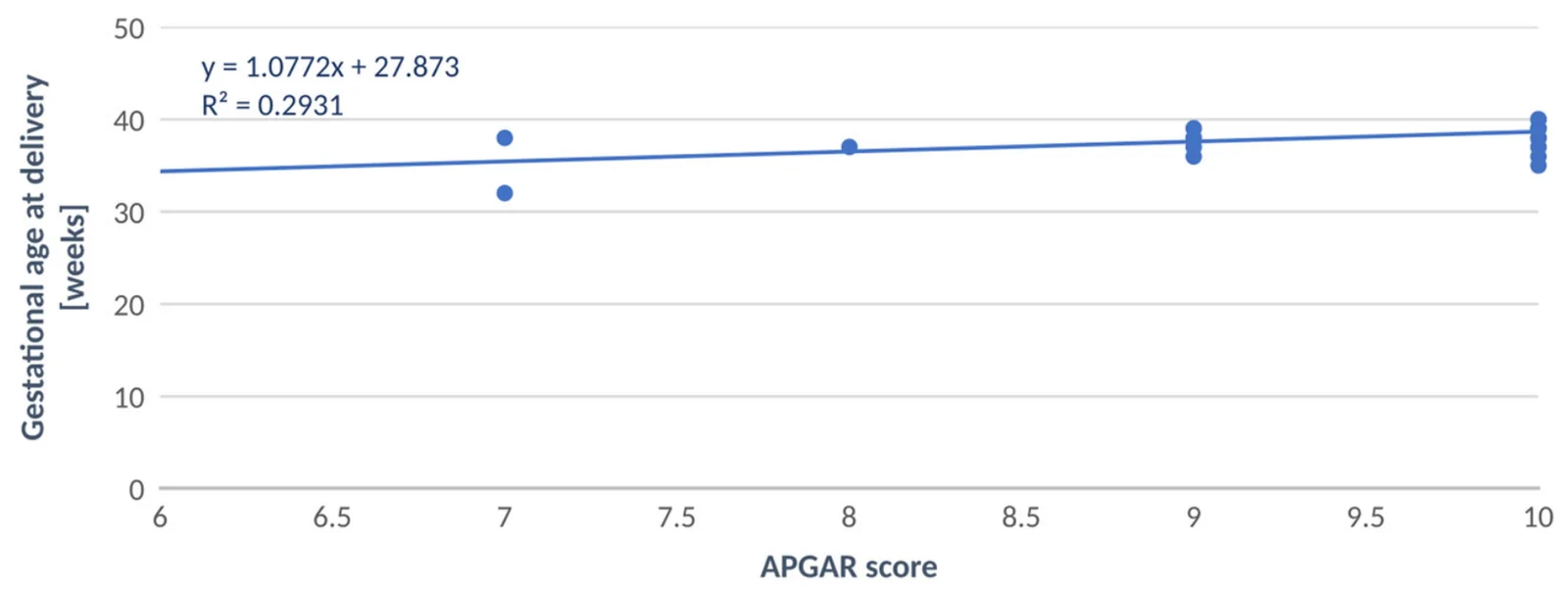
Gestational age at delivery and APGAR scores in the study group (rho = 0.438; *n* = 50; *p* = 0.001).

**Figure 7:**
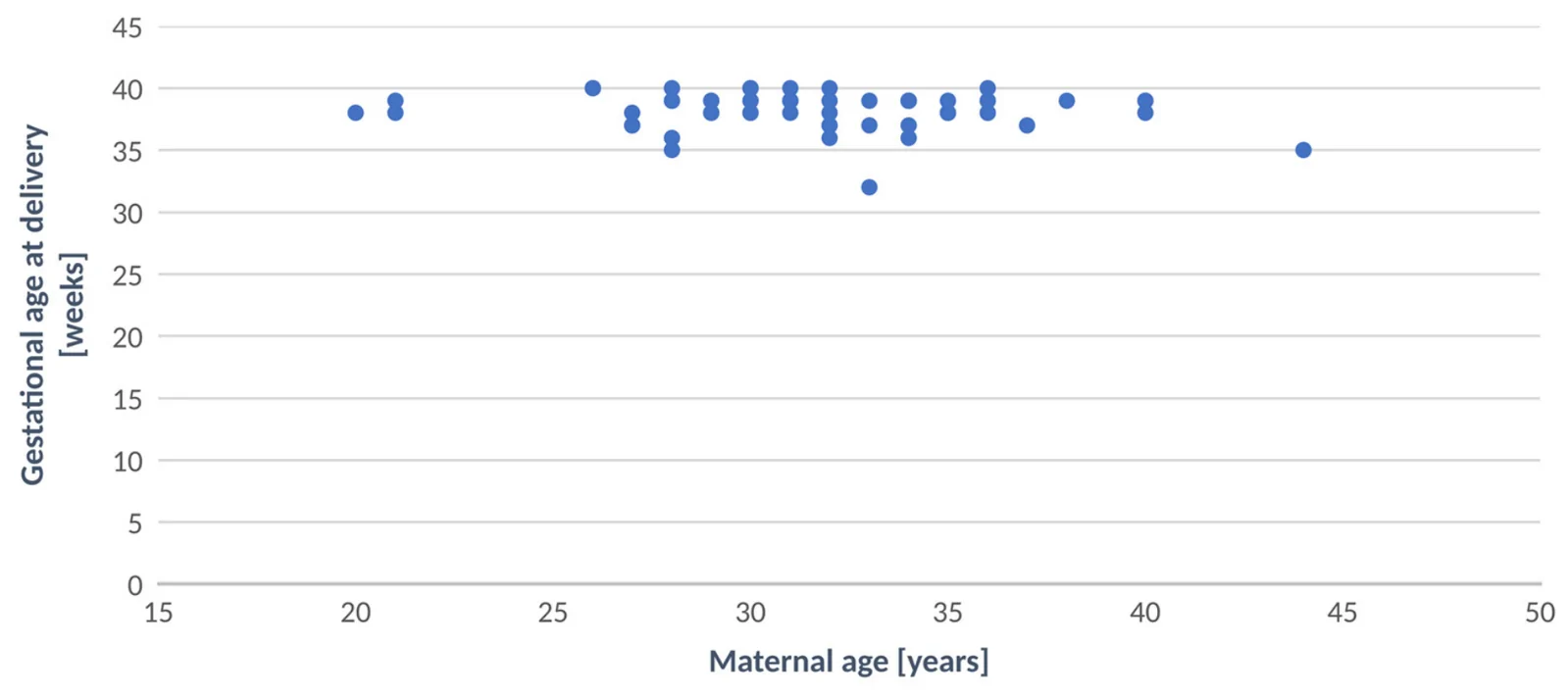
Maternal age and gestational age at delivery in the study group (rho = −0.081; *n* = 58; *p* = 0.547).

**Figure 8:**
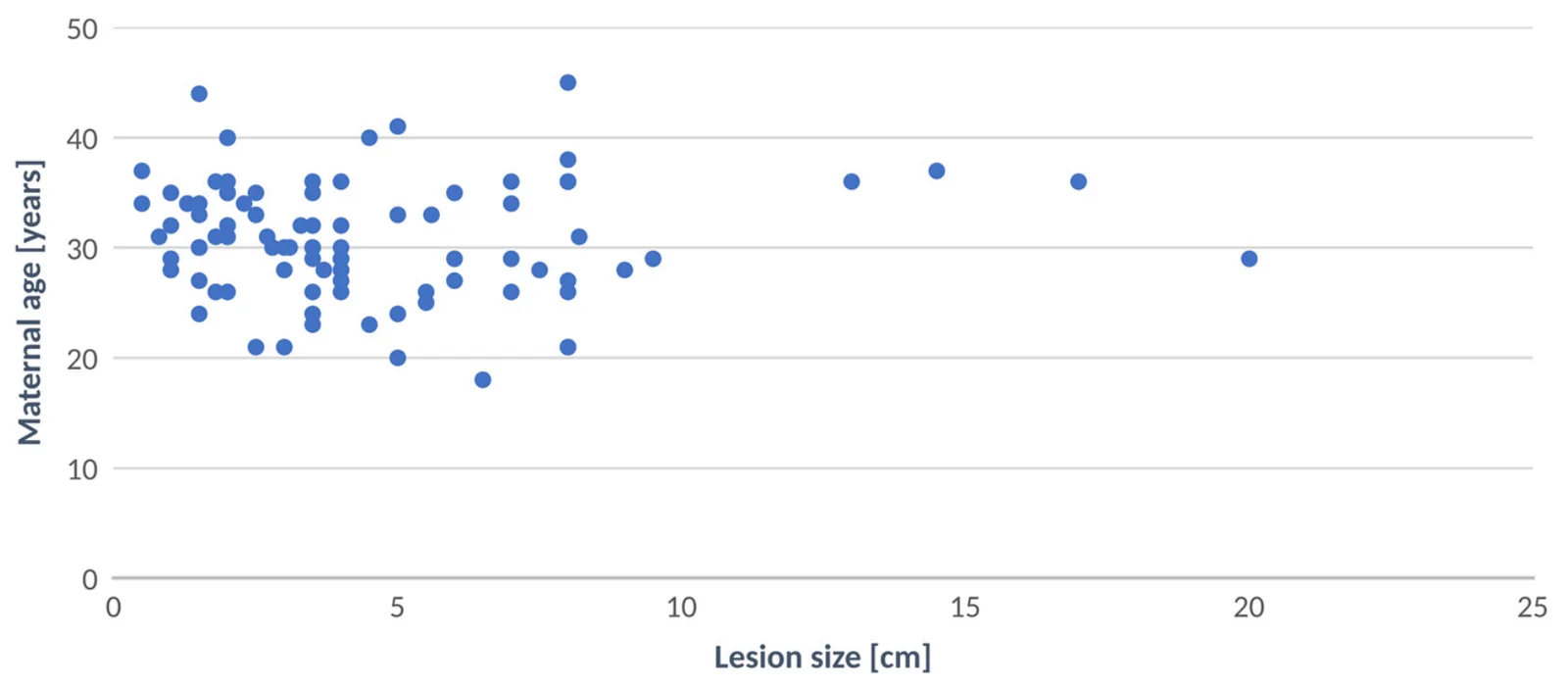
Lesion size and maternal age in the study group (rho = −0.108; *n* = 94; *p* = 0.302).

- A summary of all correlation and association analyses is presented in Table 6. Lesion size was weakly and positively correlated with the number of maternal comorbidities (rho = 0.247; *n* = 94; *p* = 0.016) (Figure 4).

- No correlation was observed between lesion size and APGAR score (rho = −0.147; *n* = 50; *p* = 0.307) (Figure 5).

- Gestational age at delivery was moderately correlated with the APGAR score (rho = 0.438; *n* = 50; *p* = 0.001) (Figure 6). This association was not adjusted for multiple comparisons and should be regarded as exploratory.

- There was no correlation between maternal age and gestational age at delivery (rho = −0.081; *n* = 58; *p* = 0.547) (Figure 7).

- There was no correlation between lesion size and maternal age (rho = −0.108; *n* = 94; *p* = 0.302) (Figure 8).

- Lesion size was associated with the time of detection (χ^2^ = 24.540, df = 6; Monte Carlo exact *p* < 0.001; Table S4): 76.7% of lesions ≤ 2.5 cm were first detected intraoperatively during the caesarean section, compared with 37.9% of lesions > 2.5–5.0 cm and 20.0% of lesions > 5.0 cm, whereas 56.7% of lesions > 5.0 cm were detected in the first trimester. The number of pregnancies was not associated with lesion size (χ^2^ = 2.892, df = 4; *p* = 0.576; Table S5).

## 4. Discussion

In the last two decades, there has been a postponement of procreation to later years, resulting in a significant increase in the percentage of pregnancies in the 30s and 40s. Several factors contribute to this trend. In addition to the influence of environmental factors, this is mainly linked to changes in previous lifestyles, including women’s involvement at educational and professional levels. The observed trend predisposes and leads to a higher incidence of a number of pathologies and complications during pregnancy that pose a risk to both the mother and child [16]. Given these factors, the literature on ovarian tumors in pregnancy is abundant in scientific journals and is mainly concerned with malignant lesions, despite the fact that they represent a small proportion of all ovarian lesions diagnosed in pregnancy. Ovarian lesions are relatively rare in pregnant women and affect approximately 1–2% of all pregnancies [17]. Most are benign, including functional lesions such as luteomas and theca-lutein cysts, which usually occur bilaterally. Ovarian lesions can cause obstetric complications and may adversely affect the developing fetus. Reported complications in pregnancies with ovarian lesions include fetal malpresentation, preterm birth, and premature rupture of the membranes [18].

This study involved 305 pregnant women, 105 of whom were diagnosed with an ovarian lesion. Several parameters were investigated to describe the maternal and neonatal outcomes in pregnancies with ovarian lesions. The patients included in the study were hospitalized in the Department of Obstetrics and Perinatology in Lublin, Poland. In the present study, the mean age of the women studied was 30.5 years. The youngest respondent was 18 years old, while the oldest was 45 years old. The average age of women in the group in which ovarian lesions were detected was 31 years. According to the literature, the nature of the ovarian lesion may correlate with age. In the study group, 102 patients (97.1%) were diagnosed with a benign lesion, while three patients (2.9%) were diagnosed with a malignant lesion. The risk of developing a malignant ovarian lesion increases with age, with the highest incidence around the sixth to seventh decade of life (approximately 60–75 years of age) [18]. According to Rubach et al. (2018), approximately 6% of the operated lesions are malignant, while the rest are benign [19]. In contrast, Wang et al. (2020) estimated that approximately 0.44–1.71 per 10,000 pregnancies of detected ovarian lesions are malignant [20].

Caesarean sections were recorded in 57 of the 58 women in the study group who delivered in the department (98.3%), compared with 48.5% in the reference group. This finding must be interpreted with considerable caution. First, no delivery was recorded in the department for 47 of 105 women (44.8%), and in the worst-case scenario, the caesarean rate in the study group was 57.6%, and no longer differed significantly from that in the reference group. Second, and more importantly, 42% of the lesions were first detected intraoperatively during caesarean sections; such lesions can, by definition, be identified only in women who undergo caesarean delivery, so the case definition itself selects caesarean deliveries into the study group. Third, the recorded indications for caesarean sections were predominantly obstetric or maternal (e.g., neurological indications, previous caesarean sections, and breech presentation) rather than lesion-related. Therefore, our data demonstrate an association between ovarian lesions and caesarean delivery, but they do not show that the ovarian lesion was the reason for operative delivery. Previous studies have also reported that caesarean delivery is more frequent in the presence of an ovarian tumor [21]. A large lesion may theoretically obstruct the birth canal or predispose to a non-cephalic presentation, and caesarean section allows concurrent removal and histopathological evaluation of the lesion and the planning of further oncological treatment when needed [22]. However, a benign ovarian lesion is not in itself an indication for caesarean delivery, and the mode of delivery should be determined by obstetric indications. In our cohort, ovarian lesions were most often identified during caesarean section or in the first trimester of pregnancy, which is consistent with the literature. Most ovarian lesions are asymptomatic or give uncharacteristic symptoms, which can make diagnosis difficult, especially when they are atypically located. In addition, the lesion may also appear later in pregnancy and may also be difficult to diagnose.

In the study conducted, the mean gestational age at delivery was 38.2 weeks in the study group and 38.8 weeks in the reference group. Although statistically significant, this difference in less than one week is of limited clinical relevance, the proportion of preterm births (10.3% vs. 4.0%) did not differ significantly, and the data could not be adjusted for potential confounders such as twin pregnancy. Preterm birth in pregnant women with an ovarian lesion may result from rupture of the lesion with associated bleeding and/or its large size; the mass, by exerting pressure on the uterine muscle and stimulating contractions, may induce preterm labor [23]. The issue of ovarian malignancies and the associated decision-making pose many problems and often evoke strong emotions. In the group of three patients diagnosed with a malignant neoplastic process, the pregnancy ended at 32 weeks + 5 days (mean value). Due to the small size of this group, it was not subjected to statistical analysis. Caesarean section was performed in this group because of the enlargement of the ovarian lesion, the suspected malignant morphological features of the tumor on ultrasound, and the complaints of pain reported by the patients. Abnormal ovarian lesions can take a variety of forms and degrees of malignancy. The management principles are based on the current clinical condition of the pregnant patient, possible available treatments, gestational age, history of chronic diseases, and hereditary factors. A key issue, especially in the early stages of cancer, is the patient’s wish to have offspring in the future [24].

The surgical treatment and its extent should be mindful of the negative consequences for the mother and fetus. Some ovarian malignancies during pregnancy require follow-up treatment. Chemotherapy, especially in the first trimester of pregnancy, can cause fetal abnormalities and pregnancy loss in some cases.

Gholson et al. (2014) reported that a large adnexal mass was associated with lower birth weight in newborns of mothers with ovarian tumors compared with children of mothers without such masses [11]. In the present study, the mean birth weight of the newborns was 3360 g in the study group and 3387 g in the reference group, and the difference was not statistically significant. This may be explained by the relatively small size of most lesions (median 3.5 cm) and the predominance of benign lesions. However, other unmeasured factors cannot be excluded.

In our cohort, endocrine, metabolic, and genitourinary diseases were more frequent among women with ovarian lesions than in the reference group, whereas the differences in cardiovascular and gastrointestinal diseases did not remain significant after correction for multiple comparisons. Similar associations have been described by other authors [25], but the genitourinary category may partly reflect the coding of the ovarian lesion itself. It cannot be ruled out that immune system dysfunctions or endocrine disorders may also be involved in the onset and progression of gonadal malignancies, as also observed by other authors [26]. The association of obesity with ovarian cancer risk is explained by increased adipocytes, which are a source of inflammatory factors that stimulate the growth of tumor cells through stimulation of the transcription activator STAT3 and the process of angiogenesis [27]. However, this association is subtype-dependent, being reported mainly for borderline, endometrioid, clear-cell, and mucinous tumors rather than for the more common high-grade serous carcinomas, and meta-analyses have reported only a slight overall correlation between BMI and ovarian cancer risk [27]. In the present study, the mean BMI was lower in the study group than in the reference group (26.4 vs. 28.2 kg/m^2^), although this difference disappeared when the analysis was restricted to women who delivered in the department (28.3 vs. 28.2 kg/m^2^). Overweight and obesity are often linked not only to geographical location, but also to the impact of the immediate environment, eating habits, and the functioning of the health system and preventive information campaigns, Overweight or obesity was present in 70.2% of all women in this study, which may be explained by a positive energy balance in the face of reduced daily physical activity and extensive access to high-calorie processed foods. Pregnant patients with oncology problems largely experience uncertainty about the future, a sense of loneliness about the situation, and a lowered mood, which can also result in a lack of desire to engage in physical activity [28]. Additionally, conditions such as atypical depression can cause increased appetite. It should be pointed out that for many years there has been a commonly held belief in Polish society that it is necessary to eat more during pregnancy, which does not correspond to the results of scientific studies recommending an increase in calories of only about 300 kcal/day during pregnancy [29]. Because the lower BMI in the whole study group reflected the earlier timing of weight measurement in women without a recorded delivery, and BMI did not differ among women who delivered in the department, our data do not support a link between obesity and the presence of ovarian lesions in pregnancy. On the other hand, it cannot be ruled out that the fat cell products mentioned above may promote tumorigenesis. An imbalance between pro-inflammatory and anti-inflammatory factors, including hormonal changes prior to pregnancy, may result in a chronic inflammatory process that may promote carcinogenesis; this hypothesis could not be tested in the present study.

An interesting aspect of this study was the blood group analysis. In both the reference and study groups, ARh+ and 0Rh+ blood groups predominated, which was not confirmed by the results obtained by Gates et al. (2011) [30]. The researchers showed that blood type B was positively correlated with the incidence of ovarian lesions, whereas blood type A was not associated with the observed lesions. Overall, the association between ABO blood group and ovarian cancer risk appears to be modest and inconsistently reported across studies, with pooled analyses showing only a slightly elevated risk for blood group A relative to group O (OR approximately 1.09–1.17) and no association consistently reported for the other groups [31,32]. An explanation for our results may be the varying distribution of different blood groups depending on the region or geographic latitude. Referring to the results of our own study and those obtained by other authors, it is difficult to unequivocally answer the question of what is the relationship between blood group and ovarian lesions. Given the modest effect sizes reported in the literature and the fact that blood group data were missing for 26 women in the study group, this finding should be regarded as hypothesis-generating rather than conclusive, particularly given the small number of malignant cases in the present cohort.

Analysis of neonatal outcomes showed that in both the study and reference groups, 96% and 98% of newborns, respectively, were born in good condition and scored 8–10 points on the APGAR scale. A small percentage of newborns scored lower (3.9% in the study group and 1.5% in the reference group received a moderate score of 4–7 points), and only 0.5% of newborns in the reference group received 0–3 points. The results obtained are consistent with those obtained previously by other researchers. Kiemtoré et al. (2019) reported an APGAR score of 8/10 points at the first minute after birth and 10/10 points at the fifth minute in a newborn born to a mother with an ovarian cyst [33]. In addition, 8/10 and 10/10 points were scored by neonates in the study by Erol et al. (2013) [34]. In the study by Sherard et al. (2003), 9% of pregnancies ended in preterm labor, with mean APGAR scores ranging from 7.5 to 8.7/10 points. High-quality obstetric care and modern treatment methods can contribute significantly to the good condition of the newborn assessed at birth [35].

The higher frequency of atrial septal defects among newborns of women with ovarian lesions (22.9% vs. 6.1%) was the only neonatal difference that remained statistically significant after correction for multiple comparisons. This finding should not be interpreted causally. Neonatal diagnoses were available for fewer than half of the newborns in the study group, and the newborns with available records may differ systematically from those without. Moreover, newborns of mothers with an ovarian lesion, and particularly those born preterm or by caesarean section, may have been more likely to undergo postnatal echocardiography, which would increase the detection of small, often physiological interatrial communications (small secundum defects, many of which close spontaneously). A biological mechanism linking a predominantly benign ovarian lesion to cardiac septation, which is completed early in the first trimester, is not apparent, and the data did not allow adjustment for potential confounders such as maternal diabetes, gestational age at birth, or twin pregnancy. Similarly, respiratory failure was more frequent in the study group, but this difference did not remain significant after FDR correction and may be related to the shorter duration of pregnancy, and the frequency of neonatal jaundice was similar in both groups. These neonatal findings are therefore hypothesis-generating and require confirmation in studies with complete and standardized neonatal assessments.

The size of the diagnosed ovarian lesions ranged from 0.5 cm to 20.0 cm. The mean and median values were 4.4 cm and 3.5 cm, respectively. In the literature, the reported mean size of adnexal masses in pregnancy is approximately 5 cm: 47.7 mm (95% CI: 39.9–55.4) according to Eichenberger-Gautschi et al. (2017) [36] and 48 mm (range, 12–115 mm) according to Condous et al. (2004) [37].

Several weak to moderate correlations and associations were observed in the study group: lesion size correlated with the number of comorbidities (rho = 0.247; *n* = 94; *p* = 0.016), gestational age at delivery correlated with the APGAR score (rho = 0.438; *n* = 50; *p* = 0.001), and the number of pregnancies was associated with postpartum anemia (*p* = 0.048). These relationships were weak to moderate, were not adjusted for multiple testing, and some (such as the association between the number of pregnancies and anemia) lacked a consistent trend; therefore, they should be regarded as hypothesis-generating. The association between lesion size and time of detection most likely reflects detection bias: large lesions are readily visualized on early pregnancy ultrasound, whereas small lesions are often missed antenatally and found incidentally at caesarean section.

Interestingly, no correlation was observed between lesion size and neonatal well-being, as assessed by the APGAR scale, or between maternal age and gestational age. The median lesion size in the current study was 3.5 cm. Lesions of this size did not appear to affect the condition of the newborns, which could be explained by the unilateral location of most lesions or by the implantation and development of the embryo in a location that did not interfere with the lesion.

The mean age of the mothers in the study and reference groups was 31 and 30 years, respectively, and the mean gestational age at delivery was 38.2 and 38.8 weeks, respectively. Therefore, maternal age is unlikely to explain the difference in the duration of pregnancy. Whether the shorter duration of pregnancy in the study group reflects the ovarian lesion itself, associated comorbidities (including genitourinary and endocrine diseases), surgical interventions, or the timing of planned delivery cannot be determined from our data.

It is worth noting the indications for caesarean sections in the group of women diagnosed with an ovarian lesion. Maternal neurological indications were the predominant indication for caesarean sections, accounting for 33% of all recorded indications. The next most frequent were previous caesarean sections (13%), breech presentation of the fetus (9%), and gestational diabetes mellitus (5%). Only 2% of the indications in this group were threatened intrauterine fetal asphyxia. These observations do not coincide with the statistically most common indications, which are lack of progress in labor and threatened intrauterine fetal asphyxia, and they suggest that a considerable proportion of caesarean sections in this group were planned for maternal reasons unrelated to the ovarian lesion.

In summary, gynecological oncology raises many issues, especially in relation to pregnant patients, where the lives of the mother and child are at risk of being compromised. Receiving the news of being diagnosed with an ovarian lesion causes pregnant women understandable anxiety and fear for their own health and that of their baby. The diagnosis of these lesions is very difficult, and treatment, which is sometimes complex, requires extensive knowledge and the involvement of different groups of specialists. In making therapeutic decisions, the well-being of the mother and child should always be considered, and therapy should be selected on a case-by-case basis. In parallel, the patient should be surrounded by medical staff who show support and empathy toward them. From the clinician’s perspective, it is crucial to analyze the factors influencing the onset of ovarian pathology to improve the diagnosis of the condition and, in the future, to gain a deeper understanding of the mechanisms responsible for the pathological changes.

Regarding the results obtained, we emphasize the importance of early diagnosis of ovarian lesions, which may contribute to better prognosis. In the case of malignant lesions, delayed diagnosis may be associated with worse prognosis. Lesions may be missed for a variety of reasons: technical aspects and the associated resolution of the equipment used, the experience of the examiner, dedicating an appropriate amount of time to the examination, focusing too much on the assessment of pregnancy while neglecting the evaluation of the ovaries, or difficulties in visualizing the ovaries, especially in the case of pre-existing pathologies or surgical treatment and altered ovarian topography. The high proportion of lesions detected only at caesarean section in our cohort supports the view that an assessment of the adnexa should be a compulsory part of the ultrasound examination in pregnancy. The key decision is always up to the pregnant woman after a thorough presentation of her clinical situation, taking into account all the advantages and disadvantages of the proposed treatment.

Our findings should be interpreted in the context of current international guidelines. The 2024 ESGO-ESMO-ESP consensus recommendations on ovarian cancer recommend that all patients with a suspicious adnexal mass during pregnancy be evaluated at a specialist referral center, with expert ultrasound as the first-line imaging modality and MRI reserved for indeterminate cases. Routine measurement of tumor markers, such as CA-125, is not recommended, as their levels are physiologically altered by pregnancy and can be misleading [38]. Where surgical intervention is indicated, it is generally best performed in the second trimester to minimize the risk of miscarriage or preterm labor, and cytotoxic chemotherapy is contraindicated in the first trimester because of the risk of major fetal malformations. In our cohort, most surgical interventions were performed at the time of the caesarean section, in line with the management pattern reported by Ritta et al. (2025) in a comparably sized retrospective series [12]. These considerations support the recommendation that pregnant women with a suspicious ovarian lesion should be managed by a multidisciplinary team at a center experienced in both maternal-fetal medicine and gynecologic oncology.

This study had several limitations. First, its retrospective, single-center design carries an inherent risk of selection and information bias and does not allow for causal inference. Second, data were missing for several important variables, particularly delivery data (44.8% of the study group) and neonatal diagnoses (54.3% of the study group); the missingness was concentrated in the study group and was unlikely to be random, which may have biased the comparisons, and the difference in the caesarean rate was no longer statistically significant in the worst-case sensitivity analysis. Third, the case definition introduced detection bias: 42% of lesions were identified only at caesarean section, which inflated the caesarean rate in the study group and, conversely, indicated that small lesions in women who delivered vaginally were probably not detected. Fourth, the ascertainment of neonatal diagnoses, including atrial septal defects, may have differed between groups. Fifth, the number of events and the extent of missing data did not allow multivariable adjustment for potential confounders such as BMI, comorbidities, gestational age, or multiple pregnancies, and residual confounding cannot be excluded. Sixth, numerous secondary comparisons were performed; although the FDR correction was applied to comorbidities and neonatal diagnoses, the remaining exploratory analyses were not adjusted and may include chance findings. Seventh, the study group was heterogeneous, including cystic and solid benign neoplasms and three malignant tumors (*n* = 3), precluding a meaningful subgroup analysis. Our findings primarily apply to benign ovarian lesions. Finally, the study population was recruited over a ten-year period (2011–2020), during which diagnostic technology and clinical practice, including the routine use of standardized ultrasound risk-stratification systems such as IOTA or O-RADS, evolved considerably; these tools were not systematically applied in the historical records reviewed. Future prospective multicenter studies with complete outcome ascertainment, standardized ultrasound risk stratification, and longer-term follow-up of both mothers and children are warranted to confirm and extend these findings.

## 5. Conclusions

In this retrospective, single-center cohort of predominantly benign ovarian lesions (97.1%), the birth weight and APGAR scores of newborns did not differ from those in the reference group. Caesarean delivery was more frequent among women with an ovarian lesion; however, this association is largely explained by the study design, in which 42% of lesions were detected intraoperatively at caesarean section, and by incomplete delivery data, and it does not indicate that the ovarian lesion itself was the indication for caesarean delivery. Ovarian lesions were most often identified at caesarean section or in the first trimester, underscoring the value of a systematic adnexal assessment as part of routine early-pregnancy ultrasound. Lesion size correlated weakly with the number of maternal comorbidities (rho = 0.247; *p* = 0.016). The higher frequency of atrial septal defect observed among newborns of women with ovarian lesions (22.9% vs. 6.1%) was based on the subset with available neonatal records and may reflect ascertainment bias; together with the other secondary findings, it should be regarded as hypothesis-generating and requires confirmation in larger, prospective, multicenter cohorts with complete outcome data. Pregnant women with a suspicious ovarian lesion should be managed individually by a multidisciplinary team involving obstetricians, gynecologic oncologists, and neonatologists, while the mode of delivery should be determined by obstetric indications.

## Figures and Tables

**Table 1:** Baseline maternal characteristics of the study and reference groups.

Characteristic	Study Group (*n* = 105)	Reference Group (*n* = 200)	*p*
**Age, years (*n* = 105/200)**			
Mean ± SD	31.0 ± 5.1	30.3 ± 5.0	0.242 ^a^
Median (Q1–Q3)	31.0 (28.0–35.0)	30.0 (27.0–34.0)	
Range	18–45	18–42	
**Height, cm (*n* = 103/200)**			
Mean ± SD	166.8 ± 5.8	166.3 ± 5.0	0.457 ^a^
Median (Q1–Q3)	167.0 (163.0–170.0)	165.5 (164.0–170.0)	
Range	150–185	152–177	
**Body weight, kg (*n* = 103/200)**			
Mean ± SD	73.1 ± 14.1	77.9 ± 12.4	0.002 ^a^
Median (Q1–Q3)	71.0 (62.1–80.8)	77.0 (69.0–85.0)	
Range	52–124	52–145	
**BMI, kg/m^2^ (*n* = 102/200)**			
Mean ± SD	26.4 ± 5.0	28.2 ± 4.1	0.001 ^a^
Median (Q1–Q3)	26.4 (22.2–29.7)	27.7 (25.3–30.1)	
Range	18.3–40.4	19.6–50.2	
**BMI category, *n* (%) (*n* = 102/200)**			**0.001 ^b^**
<25.0 kg/m^2^	44 (43.1)	46 (23.0)	
25.0–29.9 kg/m^2^	36 (35.3)	102 (51.0)	
≥30.0 kg/m^2^	22 (21.6)	52 (26.0)	
**Number of pregnancies, *n* (%) (*n* = 100/200)**			**0.632 ^b^**
1	43 (43.0)	83 (41.5)	
2	32 (32.0)	72 (36.0)	
3	14 (14.0)	31 (15.5)	
≥4	11 (11.0)	14 (7.0)	
**ABO blood group, *n* (%) (*n* = 79/199)**			**0.988 ^b^**
A	29 (36.7)	76 (38.2)	
B	16 (20.3)	38 (19.1)	
AB	5 (6.3)	14 (7.0)	
0	29 (36.7)	71 (35.7)	
Rh negative, *n* (%) (*n* = 79/199)	17 (21.5)	39 (19.6)	0.741 ^c^

Values are presented as mean ± SD, median (Q1–Q3), range, or *n* (%) of women with available data (*n*). SD, standard deviation; Q1–Q3, first–third quartile. ^a^ Student’s *t*-test; ^b^ χ^2^ test; ^c^ Fisher’s exact test. The category < 25.0 kg/m^2^ includes women with normal weight and underweight (minimum BMI 18.3 kg/m^2^). The number of pregnancies includes the current pregnancy. The detailed blood group distribution is presented in Table S1. Rows in bold indicate variable headings; *p*-values in these rows refer to the overall comparison for the variable.

**Table 2:** Maternal comorbidities in the study and reference groups (per patient analysis).

Category	Study Group (*n* = 105)	Reference Group (*n* = 192)	*p*	q
Metabolic diseases	7 (6.7)	0 (0.0)	<0.001	0.002
Cardiovascular diseases	19 (18.1)	20 (10.4)	0.073	0.097
Genitourinary diseases	105 (100.0)	106 (55.2)	<0.001	<0.001
Pulmonary diseases	0 (0.0)	7 (3.6)	0.054	0.081
Gastrointestinal diseases	7 (6.7)	3 (1.6)	0.037	0.074
Endocrine diseases	16 (15.2)	1 (0.5)	<0.001	<0.001
Immune diseases	5 (4.8)	25 (13.0)	0.026	0.063
Visual impairment/otolaryngological diseases	0 (0.0)	2 (1.0)	0.541	0.591
Musculoskeletal diseases	2 (1.9)	1 (0.5)	0.286	0.343
Previous caesarean section	17 (16.2)	16 (8.3)	0.052	0.081
Other (genetic, infectious) diseases	0 (0.0)	1 (0.5)	1.000	1.000
No comorbidity	0 (0.0)	56 (29.2)	<0.001	<0.001

Values are numbers (%) of women with at least one diagnosis in the respective category; a woman could have diagnoses in more than one category. Comorbidity data were missing for 8 women in the reference group. *p*, Fisher’s exact test; q, Benjamini–Hochberg false discovery rate-adjusted *p*-value (12 comparisons).

**Table 3:** Characteristics of ovarian lesions in the study group.

Characteristic	Study Group (*n* = 105)
** Lesion size, cm (*n* = 94)**	
Mean ± SD	4.4 ± 3.4
Median (Q1–Q3)	3.5 (2.0–6.0)
Range	0.5–20.0
** Lesion size category, *n* (%) (*n* = 94)**	
≤2.5 cm	30 (31.9)
>2.5–5.0 cm	32 (34.0)
>5.0 cm	32 (34.0)
** Time of detection, *n* (%) (*n* = 100)**	
First trimester (≤14 weeks)	41 (41.0)
Second trimester (15–27 weeks)	13 (13.0)
Third trimester (≥28 weeks)	4 (4.0)
Intraoperatively during caesarean section	42 (42.0)
** Histopathology, *n* (%) (*n* = 105)**	
Benign (ICD-10 D27)	102 (97.1)
Malignant (ICD-10 C56)	3 (2.9)
Histological type, n	Benign: simple cyst 89, mature cystic teratoma 8, endometriotic cyst 5; malignant: mucinous adenocarcinoma 2, serous carcinoma 1

Values are presented as mean ± SD, median (Q1–Q3), range, or *n* (%) of women with available data (*n*). Variable headings are shown in bold.

**Table 4:** Pregnancy and delivery outcomes in the study and reference groups.

Outcome	Study Group (*n* = 105)	Reference Group (*n* = 200)	Effect (95% CI)	*p*
**Gestational age at delivery, weeks (*n* = 58/198)**				
Mean ± SD	38.2 ± 1.5	38.8 ± 1.6	−0.6 (−1.0 to −0.2) ^a^	0.009
Median (Q1–Q3)	39.0 (38.0–39.0)	39.0 (38.0–40.0)		
Range	32–40	27–41		
Preterm birth < 37 + 0 weeks, *n* (%)	6 (10.3)	8 (4.0)	RR 2.56 (0.93–7.08) ^b^	0.094
Very preterm birth < 32 + 0 weeks, *n* (%)	0 (0.0)	2 (1.0)		
Extremely preterm birth < 28 + 0 weeks, *n* (%)	0 (0.0)	1 (0.5)		
Pregnancy loss < 22 weeks, *n* (%)	6/105 (5.7)	0 (0.0)		
**Mode of delivery, *n* (%) (*n* = 58/200)**				
Vaginal	1 (1.7)	103 (51.5)		
Caesarean section	57 (98.3)	97 (48.5)	RR 2.03 (1.75–2.35) ^b^	<0.001
No delivery recorded in the department	47 (44.8 of 105)	0		
**Sensitivity analysis: caesarean section**				
Worst-case scenario (*n* = 99/200)	57 (57.6)	97 (48.5)	RR 1.19 (0.95–1.48) ^b^	0.143
Best-case scenario (*n* = 99/200)	98 (99.0)	97 (48.5)	RR 2.04 (1.77–2.36) ^b^	<0.001
Antenatally detected lesions only	15/16 (93.8)	97 (48.5)	RR 1.93 (1.60–2.34) ^b^	<0.001
Postpartum anemia (Hb ≤ 11 g/dL), *n* (%)	43/94 (45.7)	not analyzed		

Values are presented as mean ± SD, median (Q1–Q3), range, or n (%) of women with available data (*n*). CI, confidence interval; RR, relative risk. ^a^ Welch’s *t*-test (mean difference, 95% CI); Mann–Whitney U test *p* < 0.001; ^b^ Fisher’s exact test (RR, 95% CI). Gestational age at delivery was analyzed in women who delivered in the department. Worst-case scenario: all women with an ongoing pregnancy but no recorded delivery (*n* = 41) were assumed to have delivered vaginally; best-case scenario: all were assumed to have delivered by caesarean section; women with a pregnancy loss before 22 weeks (*n* = 6) were excluded. Pregnancy loss comprised three miscarriages and three tubal ectopic pregnancies. Variable headings are shown in bold.

**Table 5:** Neonatal outcomes in the study and reference groups.

Outcome	Study Group (*n* = 105)	Reference Group (*n* = 200)	Effect (95% CI)	*p*	q
** Birth weight, g (*n* = 58/200)**					
Mean ± SD	3360 ± 530	3387 ± 445	−27 (−164 to 109) ^a^	0.696	
Median (Q1–Q3)	3400 (3090–3658)	3405 (3123–3670)			
Range	1980–4500	1220–4360			
** APGAR category, *n* (%) (*n* = 51/200)**				**0.418 ^b^**	
Poor (0–3)	0 (0.0)	1 (0.5)			
Moderate (4–7)	2 (3.9)	3 (1.5)			
Good (8–10)	49 (96.1)	196 (98.0)			
** Neonatal diagnoses, *n* (%) (*n* = 48/198) ^c^**			**RR (95% CI)**		
Atrial septal defect	11 (22.9)	12 (6.1)	3.78 (1.78–8.04)	0.001	0.022
Respiratory failure of newborn	5 (10.4)	4 (2.0)	5.16 (1.44–18.48)	0.016	0.100
Other respiratory disturbances of newborn	1 (2.1)	8 (4.0)	0.52 (0.07–4.02)	1.000	1.000
Infant of mother with gestational diabetes	4 (8.3)	1 (0.5)	16.50 (1.89–144.30)	0.006	0.053
Other cases of prematurity	4 (8.3)	7 (3.5)	2.36 (0.72–7.73)	0.233	0.491
Neonatal jaundice, unspecified	9 (18.8)	34 (17.2)	1.09 (0.56–2.12)	0.833	0.989
Neonatal jaundice associated with preterm delivery	1 (2.1)	1 (0.5)	4.12 (0.26–64.78)	0.353	0.559
Infection specific to the perinatal period	2 (4.2)	1 (0.5)	8.25 (0.76–89.11)	0.098	0.311
Persistent fetal circulation	2 (4.2)	2 (1.0)	4.12 (0.60–28.55)	0.172	0.409
ABO isoimmunisation of fetus and newborn	2 (4.2)	1 (0.5)	8.25 (0.76–89.11)	0.098	0.311

Values are expressed as mean ± SD, median (Q1–Q3), range, or *n* (%) of newborns with available data (*n*). CI, confidence interval; RR, relative risk; q, Benjamini–Hochberg false discovery rate-adjusted *p*-value across all 19 clinical neonatal diagnoses (Table S3). ^a^ Student’s *t*-test (mean difference, 95% CI); ^b^ Fisher–Freeman–Halton exact test; ^c^ Fisher’s exact test. A newborn could have more than one diagnosis. Rows in bold indicate variable headings; *p*-values in these rows refer to the overall comparison for the variable.

**Table 6:** Summary of correlation and association analyses in the study group.

Variables	Test	*n*	Statistic	*p*
Lesion size × number of comorbidities	Spearman	94	rho = 0.247	0.016
Lesion size × APGAR score	Spearman	50	rho = −0.147	0.307
Gestational age at delivery × APGAR score	Spearman	50	rho = 0.438	0.001
Maternal age × gestational age at delivery	Spearman	58	rho = −0.081	0.547
Lesion size × maternal age	Spearman	94	rho = −0.108	0.302
Number of pregnancies × postpartum anemia	χ^2^	94	χ^2^ = 6.092; df = 2	0.048
Lesion size category × postpartum anemia	χ^2^	90	χ^2^ = 0.722; df = 2	0.697
Lesion size category × time of detection	χ^2^ (Monte Carlo)	89	χ^2^ = 24.540; df = 6	<0.001
Number of pregnancies × lesion size category	χ^2^	90	χ^2^ = 2.892; df = 4	0.576

rho, Spearman’s rank correlation coefficient; χ^2^, chi-square test. Gestational age at delivery in completed weeks (women who delivered in the department). None of the exploratory analyses were adjusted for multiple comparisons.

## Data Availability

The data presented in this study are available on request.

## Supplementary Materials

The following supporting information can be downloaded at: https://www.mdpi.com/article/10.3390/cancers18213463/s1, Table S1: Blood groups in the study and reference groups; Table S2: Distribution of APGAR scores; Table S3: Neonatal diagnoses in the study and reference groups; Table S4: Lesion size and time of detection; Table S5: Number of pregnancies and lesion size.
