# Supplementary material for: Maternal and Perinatal Outcomes in Pregnancies Complicated by Predominantly Benign Ovarian Lesions: A Retrospective Case-Control Study of 305 Pregnancies
Source: Cancers (Basel). 2026 Oct 27;18(21):3463. doi: 10.3390/cancers18213463 (PMC13761504; doi:10.3390/cancers18213463)
Supplement: Supplementary file 1 [file cancers-18-03463-s001.zip › cancers-4616472-supplementary.pdf]

cancers

MDPI

Supplementary Materials

Maternal and Perinatal Outcomes in Pregnancies Complicated by Predominantly Benign Ovarian Lesions: A Retrospective Case-Control Study of 305 Pregnancies

Table S1. Blood groups in the study and reference groups.

Blood group	Study group (n=79)	Reference group (n=199)	All (n=278)
ABRh−	1 (1.3)	3 (1.5)	4 (1.4)
ABRh+	4 (5.1)	11 (5.5)	15 (5.4)
ARh−	5 (6.3)	15 (7.5)	20 (7.2)
ARh+	24 (30.4)	61 (30.7)	85 (30.6)
BRh−	4 (5.1)	9 (4.5)	13 (4.7)
BRh+	12 (15.2)	29 (14.6)	41 (14.7)
0Rh−	7 (8.9)	12 (6.0)	19 (6.8)
0Rh+	22 (27.8)	59 (29.6)	81 (29.1)

Values are n (%). Data were missing for 26 women in the study group and 1 woman in the reference group. χ^2=0.938, df=7; p=0.996.

Table S2. Distribution of APGAR scores in newborns of the study and reference groups.

APGAR score	Study group (n=51)	Reference group (n=200)	All (n=251)
1	0 (0.0)	0 (0.0)	0 (0.0)
2	0 (0.0)	0 (0.0)	0 (0.0)
3	0 (0.0)	1 (0.5)	1 (0.4)
4	0 (0.0)	0 (0.0)	0 (0.0)
5	0 (0.0)	1 (0.5)	1 (0.4)
6	0 (0.0)	0 (0.0)	0 (0.0)
7	2 (3.9)	2 (1.0)	4 (1.6)
8	2 (3.9)	11 (5.5)	13 (5.2)
9	8 (15.7)	27 (13.5)	35 (13.9)
10	39 (76.5)	158 (79.0)	197 (78.5)

Values are n (%). APGAR scores were available for 51 newborns in the study group, all delivered in the department (including two twin pairs).

Table S3. Neonatal diagnoses in the study and reference groups.

Diagnosis (ICD-10, neonatal records)	Study group (n=48)	Reference group (n=198)	RR (95% CI)	p	q
Other cases of prematurity	4 (8.3)	7 (3.5)	2.36 (0.72–7.73)	0.233	0.491
Other cardiovascular disorders during perinatal period	1 (2.1)	8 (4.0)	0.52 (0.07–4.02)	1.000	1.000
Other respiratory disturbances of newborn	1 (2.1)	8 (4.0)	0.52 (0.07–4.02)	1.000	1.000
ABO isoimmunisation of fetus and newborn	2 (4.2)	1 (0.5)	8.25 (0.76–89.11)	0.098	0.311
Subgaleal haemorrhage due to	1 (2.1)	1 (0.5)	4.12 (0.26–64.78)	0.353	0.559

birth injury					
Anaemia of prematurity	2 (4.2)	2 (1.0)	4.12 (0.60–28.55)	0.172	0.409
Respiratory failure of newborn	5 (10.4)	4 (2.0)	5.16 (1.44–18.48)	0.016	0.100
Neonatal conjunctivitis and dacryocystitis	1 (2.1)	3 (1.5)	1.37 (0.15–12.93)	0.583	0.738
Persistent fetal circulation	2 (4.2)	2 (1.0)	4.12 (0.60–28.55)	0.172	0.409
Fetus/newborn affected by maternal infectious and parasitic diseases	2 (4.2)	1 (0.5)	8.25 (0.76–89.11)	0.098	0.311
Atrial septal defect	11 (22.9)	12 (6.1)	3.78 (1.78–8.04)	0.001	0.022
Moderate or mild birth asphyxia	1 (2.1)	3 (1.5)	1.37 (0.15–12.93)	0.583	0.738
Intra-amniotic infection of fetus	1 (2.1)	4 (2.0)	1.03 (0.12–9.02)	1.000	1.000
Congenital pneumonia, unspecified	1 (2.1)	2 (1.0)	2.06 (0.19–22.28)	0.480	0.702
Congenital absence and hypoplasia of umbilical artery	1 (2.1)	1 (0.5)	4.12 (0.26–64.78)	0.353	0.559
Infection specific to the perinatal period	2 (4.2)	1 (0.5)	8.25 (0.76–89.11)	0.098	0.311
Infant of mother with gestational diabetes	4 (8.3)	1 (0.5)	16.50 (1.89–144.30)	0.006	0.053
Neonatal jaundice associated with preterm delivery	1 (2.1)	1 (0.5)	4.12 (0.26–64.78)	0.353	0.559
Neonatal jaundice, unspecified	9 (18.8)	34 (17.2)	1.09 (0.56–2.12)	0.833	0.989
Live birth in hospital (Z38) *	19 (39.6)	115 (58.1)	—	—	—
Low birth weight for gestational age *	1 (2.1)	11 (5.6)	—	—	—
Other	2 (4.2)	10 (5.1)	—	—	—

Values are n (%) of newborns with available neonatal records (study group: 48 of the 58 women who delivered in the department; reference group: 198/200). A newborn could have more than one diagnosis. RR, relative risk (Haldane correction for zero cells); p, Fisher's exact test; q, Benjamini–Hochberg false discovery rate-adjusted *p*-value across the 19 clinical diagnoses. * Not included in the statistical comparison: Z38 codes denote the place of birth (live birth in hospital) rather than a disease; low birth weight for gestational age was not among the predefined clinical diagnoses.

Table S4. Lesion size and time of detection in the study group.

Time of detection	≤2.5 cm (n=30)	>2.5–5.0 cm (n=29)	>5.0 cm (n=30)
First trimester	4 (13.3)	13 (44.8)	17 (56.7)
Second trimester	2 (6.7)	3 (10.3)	7 (23.3)
Third trimester	1 (3.3)	2 (6.9)	0 (0.0)
Intraoperatively during caesarean section	23 (76.7)	11 (37.9)	6 (20.0)

Values are n (%) within each size category. χ^2=24.540, df=6; Monte Carlo exact p<0.001 (100,000 samples); 6 of 12 expected cell counts were <5. In the original submission, the intraoperative category was labelled ‘IV trimester’.

Table S5. Number of pregnancies and lesion size in the study group.

Number of pregnancies	≤2.5 cm (n=29)	>2.5–5.0 cm (n=31)	>5.0 cm (n=30)
1	15 (51.7)	11 (35.5)	12 (40.0)
2	7 (24.1)	13 (41.9)	9 (30.0)
≥3	7 (24.1)	7 (22.6)	9 (30.0)

Values are n (%) within each size category. χ^2=2.892, df=4; p=0.576.
